# Irradiance dataset in the south of Colombia from 2013 to 2023 in 5-minutes intervals

**DOI:** 10.1016/j.dib.2025.112063

**Published:** 2025-09-13

**Authors:** John Barco-Jiménez, Daniel Rosero, Andrés Zambrano, Francisco Eraso-Checa, Miller Ruales, José Camilo Eraso

**Affiliations:** a Universidad CESMAG, CRA 20A 14 54, Pasto, Nariño, Colombia; b Universidad Nacional Abierta y a Distancia, Calle 14 # 28 - 45, Sector Bomboná, Pasto, Nariño, Colombia

**Keywords:** Meteorological station data, Photovoltaic system design, Solar radiation monitoring, Renewable energy forecasting

## Abstract

This article presents an extensive irradiance dataset collected in San Juan de Pasto, located in southern Colombia, using a Davis Vantage PRO 2 meteorological station. The dataset spans 11 years, covering the period from 2013 to 2023, with measurements taken at 5-minute intervals, resulting in approximately 603,495 irradiance records, each accompanied by a corresponding timestamp.

The construction of the dataset required a rigorous preprocessing stage. This stage included the removal of erroneous values (NaN) and outliers, the identification of missing entries, and the correction of inconsistencies in the date records. Missing values were addressed through gap-filling procedures based on averaged data, complemented by visual inspections using graphical representations. The cleaned dataset was exported after ensuring data integrity, accuracy, and consistency, which are essential for reliable analysis and subsequent modeling.

This dataset is valuable for building training datasets used as input for artificial intelligence models to perform short-, medium-, and long-term irradiance forecasting. For instance, Barco-Jiménez et al. (2021) utilized a portion of this dataset to develop multitemporal irradiance predictions. These predictive models can be applied in various domains, including energy management, grid optimization, and solar energy production planning. Furthermore, the dataset supports statistical analyses that provide insights for appropriately sizing photovoltaic systems through indicators such as Hours of Peak Sunlight (HPS), maximum and minimum irradiance values, average daily and monthly irradiance, and seasonal trends. These indicators play a fundamental role in the optimization of photovoltaic system performance, contributing to cost reduction and enhancing energy efficiency across rural, residential, and commercial applications.

This dataset supports photovoltaic system design and studies on solar energy variability and climate patterns in the region. Analysis of irradiance fluctuations over time provides insights into the influence of atmospheric conditions on solar energy availability. This information is essential for enhancing the reliability of solar power systems and effectively integrating renewable energy sources into existing power grids. The dataset can also be used in educational settings to teach data analysis techniques and renewable energy concepts, providing students and researchers with a practical resource for hands-on learning.

Specifications Table

**Table:** 

Subject	Engineering & Materials science
Specific subject area	Renewable energy and global horizontal irradiance
Type of data	Table, Processed
Data collection	The data were collected in San Juan de Pasto, Colombia using a Davis Vantage PRO 2 meteorological station. The station is equipped with a silicon pyranometer for global solar radiation (SKU6450), along with sensors for air temperature (accuracy ±0.5 °C) and relative humidity (accuracy ±3 %). Solar irradiance measurements were taken at 5-minute intervals and logged continuously from January 2013 to December 2023. This produced approximately 603,495 records. Data were stored digitally on the station's console and periodically downloaded using WeatherLink software to ensure long-term preservation and prevent losses. According to the manufacturer's recommendations, preventive maintenance includes sensor cleaning and calibration checks every year. Preprocessing involved removing NaN values, physically implausible irradiance readings, and inconsistent timestamps. The identification of outliers was conducted in accordance with established statistical thresholds and through a meticulous visual inspection process. Missing values were addressed by applying average-based interpolation between neighboring observations, a procedure that preserved both short-term variability and seasonal trends. The final dataset was exported in a standardized format for further analysis and modeling.
Data source location	Institution: Universidad CESMAG City: San Juan de Pasto, Nariño, Colombia Country: Colombia Latitude and longitude for collected samples/data: 1°12′33″ N, 77°16′52″ W
Data accessibility	Repository name: Irradiance dataset in the south of Colombia from 2013 to 2023 in 5-minutes intervals Data identification number: 10.17632/hr8jgbhm5j.1 Direct URL to data: https://data.mendeley.com/datasets/hr8jgbhm5j/1 Available in [1] as “Irradiance dataset in the south of Colombia from 2013 to 2023 in 5-minutes intervals”, Mendeley Data, V1, doi:10.17632/hr8jgbhm5j.1
Related research article	A sample of the data was used in [2]: J. Barco-Jiménez, F. Eraso Checa, A. Pantoja, and E. Caicedo Bravo, “Estimation of Global Solar Radiation Using NNARX Neural Networks Based on the UV Index,” Tecnura, vol. 25, no. 70, pp. 90–107, 2021. [Online]. Available: https://doi.org/10.14483/22487638.18638

## 1 Value of the Data

• This dataset provides 11 years of irradiance measurements collected at 5-minute intervals in San Juan de Pasto City, Colombia, yielding a comprehensive time series with over 1.1 million data points. Such a rich dataset can support valuable research and applications in renewable energy, climate studies, and beyond.
• The comprehensive dataset spanning 11 years provides valuable insights into long-term trends, seasonal patterns, and the influence of atmospheric conditions on solar energy availability. This information is essential for sizing and design of photovoltaic systems.
• The dataset has undergone thorough preprocessing to ensure data integrity, accuracy, and consistency, making it a reliable resource for scientific analysis and modeling. This preprocessing involved removing erroneous values, identifying missing data, and filling gaps with averaged data.
• The dataset can serve as a valuable input for training and validating artificial intelligence models used for short-, medium-, and long-term irradiance forecasting. These predictive models have widespread applications in energy management, grid optimization, and solar energy production planning.

## 2 Background

The motivation behind compiling this comprehensive irradiance dataset was to provide a resource that could support a wide range of research and applications related to solar energy, climate studies, and renewable energy integration [[3], [4], [5]]. The region of southern Colombia, where the data was collected, has significant potential for solar energy development, but the lack of high-quality, long-term irradiance data has hindered the optimization of photovoltaic systems and the planning of solar energy projects [6].

To address this gap, a meteorological station was installed in San Juan de Pasto City to continuously monitor solar irradiance at 5-minute intervals over an 11-year period, from 2013 to 2023. The resulting dataset captures the variability and trends in solar irradiance, which are crucial for understanding the feasibility and performance of solar energy systems in the region.

## 3 Data Description

**Fig. 1:**
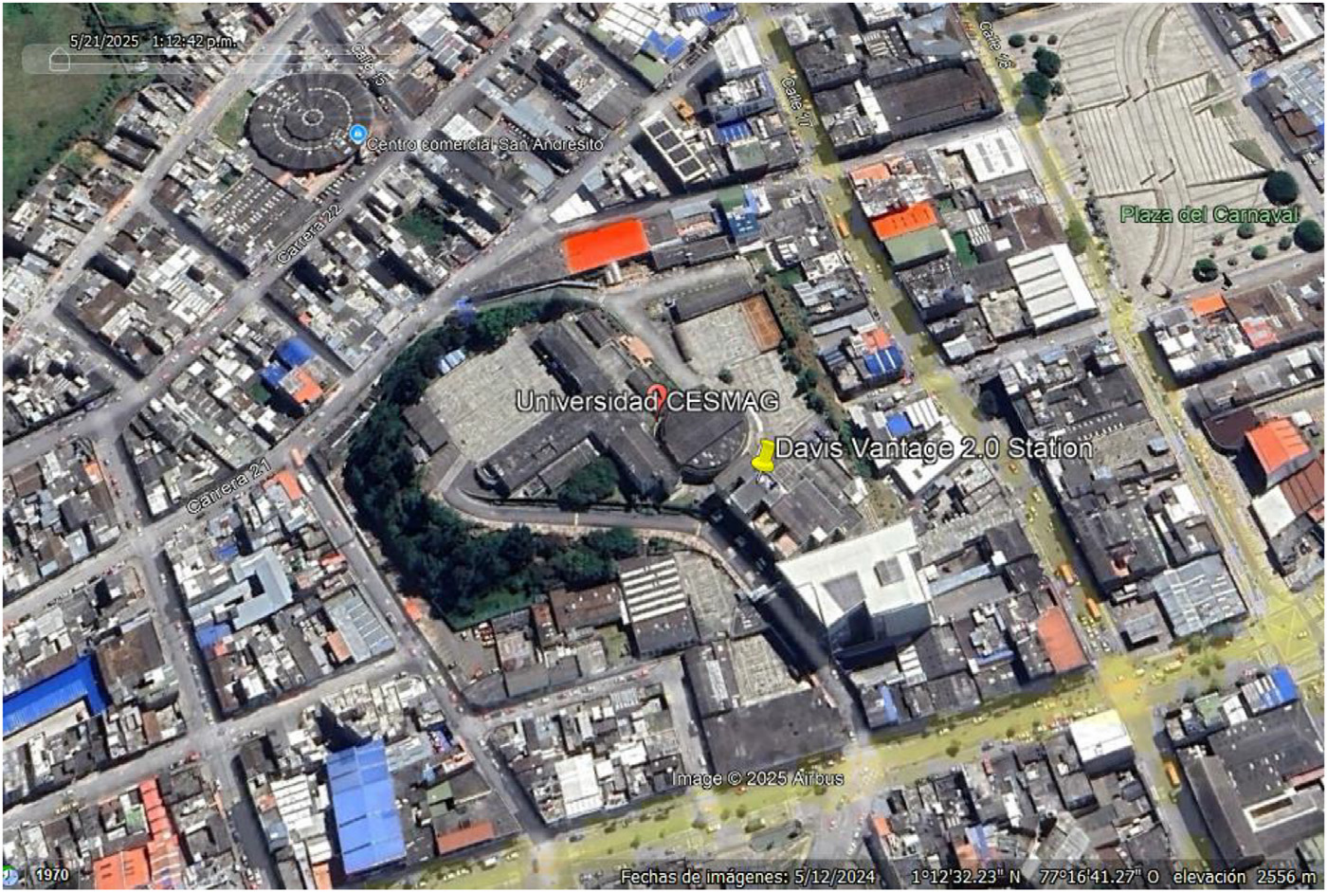
Location of the weather station (Universidad CESMAG, Pasto, Colombia).

The dataset is organized in a single Excel file named “Daily irradiance data 2013 – 2023.xls”. This file contains high-resolution daily records of global horizontal solar irradiance (GHI) measured in Watts per square meter (w/m2). Data was collected at 5-minute intervals, allowing for detailed temporal analysis. Fig. 1 shows the location of Universidad CESMAG in San Juan de Pasto, Nariño, Colombia (1°12′33″ N, 77°16′52″ W), where the weather station used for data collection is installed.

The Excel workbook comprises twelve distinct worksheet tabs, each corresponding to a specific calendar month. The data distribution across these tabs is as follows:

• Tabs “January” to “July”: These sheets contain daily irradiance data from January to July, for the years 2014 to 2023.
• Tabs “August” to “December”: These sheets contain daily irradiance data from August to December, for the years 2013 to 2023.

Each monthly tab follows a consistent structure, including the following columns:

• “Hour”: Represents the specific hour to which the irradiance data corresponds (e.g., HH:MM AM/PM).
• “Day”: Indicates the numerical day of the month (e.g., 1 to 31).
• “Year Columns (2013 - 2023)”: These columns, labeled by year, contain the irradiance data in W/m2 recorded every five minutes. Each cell in these columns corresponds to a 5-minute interval for a specific date and year.

### 3.1 File structure

Table 1 provides a summary of the data available in each monthly tab of the “Daily irradiance data 2013 – 2023.xls” file, including the range of years covered and the total number of 5-minute irradiance records per tab.

**Table 1:** Daily irradiance data 2013 – 2023.

Tabs/File	Years	Data
January	2014 - 2023	53,537
February	2014 – 2023	45,530
March	2014 – 2023	48,670
April	2014 – 2023	47,100
May	2014 - 2023	48,670
June	2014 - 2023	47,099
July	2014 - 2023	48,668
August	2013 to 2023	53,537
September	2013 to 2023	51,800
October	2013 to 2023	53,537
November	2013 to 2023	51,810
December	2013 to 2023	53,537
Total		603,495

**Fig. 2:**
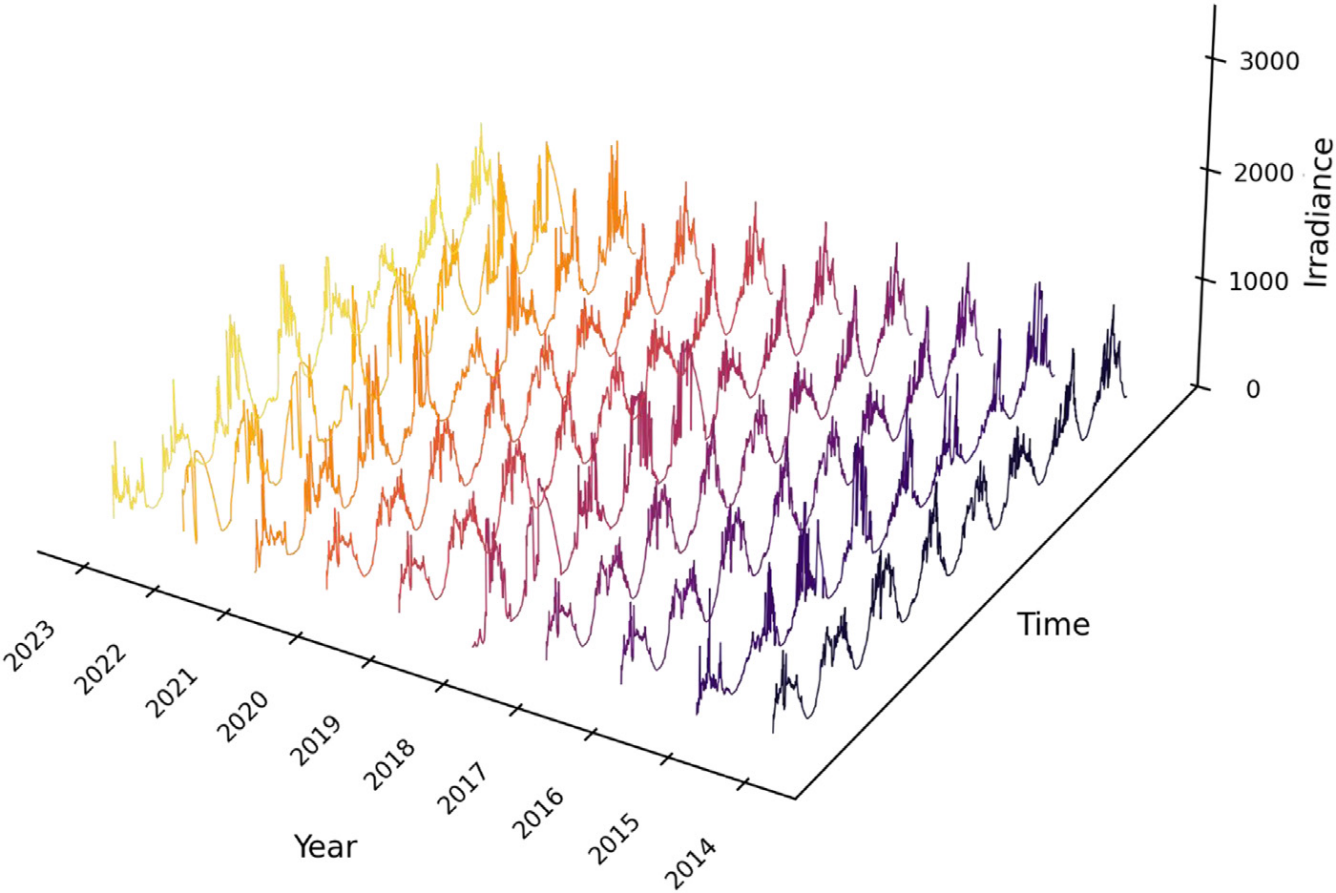
Sample irradiance behaviour over 8 days.

Fig. 2 illustrates typical daily patterns of solar irradiance from a sample of the dataset, showcasing its behavior over an eight-day period.

## 4 Experimental Design, Materials and Methods

This section provides a comprehensive description of the experimental design, instrumentation, and methodologies employed for the acquisition and initial processing of the solar irradiance data.

### 4.1 Data acquisition and measurement system

This subsection details the specific instrumentation and setup used to measure solar irradiance.

• **Measurement location:** Data were acquired at the San Francisco Laboratory located at Holanda Building at the Univesity CESMAG, in San Juan de Pasto, Nariño, Colombia with coordinates 1°12′33″ N, 77°16′52″ W; altitude 2556 m a.s.l.). The site is located in a tropical high-altitude valley with frequent cloud cover, a factor that significantly affects irradiance variability.
• **Instrumentation:** A Davis Vantage Pro 2 weather station equipped with a SKU6450 silicon pyranometer (spectral response: 400–1100 nm; sensitivity: 0.125 mV/W·m⁻²; accuracy: ±5 %) was used for global solar radiation measurements. The station also included sensors for air temperature (accuracy ±0.5 °C) and relative humidity (accuracy ±3 %).
• **Data logger and storage:** The weather station was connected to a WeatherLink data logger, which automatically recorded irradiance values at 5-minute intervals. Data were continuously logged to the internal console memory and exported monthly to a computer hard disk using WeatherLink software. Backup copies were maintained on an external hard drive to prevent data loss.
• **Calibration:** The pyranometer was by factory-calibrated against a secondary standard which is calibrated periodically against an Eppley Precision Spectral Pyranometer in natural daylight.

### 4.2 Experimental esign

• **Sensor Placement and Orientation:** The pyranometer was installed horizontally on a level platform, free from obstructions, ensuring an unobstructed 180-degree field of view.
• **Environmental Controls/Conditions:** The data was collected under ambient outdoor conditions typical of a tropical high-altitude climate at an altitude of 2556 m above sea level. This region experiences strong variability due to cloudiness and seasonal rainfall patterns, factors reflected in the dataset.

### 4.3 Data processing and quality control

**Fig. 3:**
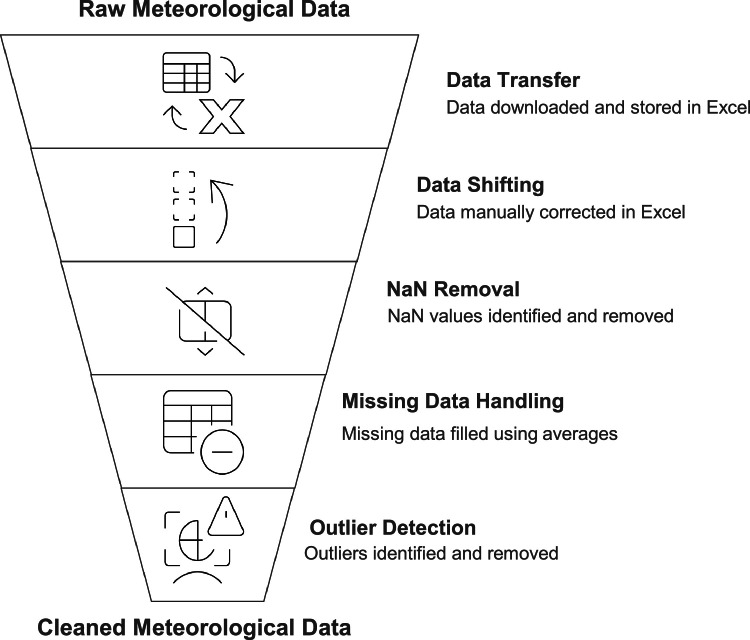
Data quality control.

All quality control steps described in Fig. 3 were systematically applied to the entire dataset covering the period 2013–2023, ensuring consistency and reproducibility of the cleaned data.

• **Initial data transfer/download:** Raw files were downloaded monthly in CSV and Microsoft Excel formats. Timestamps followed local time (GMT-5) without daylight saving adjustments.
• **Data shifting:** Occasional timestamp misalignments detected through visual inspection were manually corrected to maintain chronological consistency, with midday aligned to the maximum irradiance value.
• **NaN:** Not-a-Number mark was identified by software and erased from the table generating a missing data.
• **Missing data handling:** Gaps in the dataset, mostly due to power outages or maintenance, were identified visually and by comparing record counts with expected totals. Missing values were imputed using average-based interpolation between the same time interval of the previous and following years.
• **Outlier detection/removal:** Data underwent an initial quality control check to identify and remove outliers, including nighttime irradiance values (negative or significantly above zero) and values exceeding the maximums for the location (above 1500 W/m^2^).
• **Software for processing:** Data processing and quality control were performed using Microsoft Excel 2016, Python 3.10 with Pandas library v1.5.0, and MATLAB R2022a. The code for visualization and data pre-processing is available in [7] with link: https://github.com/johnbarco/Irradiance_dataset_2013_2023. Python libraries utilized include **Pandas** for data manipulation and handling, **NumPy** for numerical operations, **Matplotlib** for data visualization, and **Seaborn** for enhanced plots and color palettes. [visualizing_irradiance_3d.py] was used to generate Fig. 1, and [correct_missing_data.py] to complete missing data.
• **Final dataset**: The cleaned dataset was exported in CSV format, with standardized columns: *Timestamp (YYYY-MM-DD hh:mm:ss, GMT-5)* and *Global Irradiance (W/m²)*.

## Limitations

This dataset has two main limitations. First, sensor downtimes and power outages led to data gaps that were filled using averaged values from adjacent periods. While this maintains continuity, it may smooth out short-term irradiance fluctuations, underrepresenting extreme events. Second, the data were collected at a single high-altitude urban site, limiting spatial generalizability. Irradiance conditions in nearby rural or lower-altitude areas may differ significantly, so caution is advised when extending findings to broader regions.

## Ethics Statement

The presented data involved none of the following: human subjects, animal experiments, or data collected from social media platforms

## CRediT authorship contribution statement

**John Barco-Jiménez:** Conceptualization, Methodology, Data curation, Writing – review & editing. **Daniel Rosero:** Data curation, Visualization, Software. **Andrés Zambrano:** Data curation, Visualization, Software. **Francisco Eraso-Checa:** Conceptualization, Methodology, Data curation, Writing – review & editing. **Miller Ruales:** Validation, Supervision. **José Camilo Eraso:** Validation, Supervision.

## Data Availability

Mendeley DataIrradiance dataset in the south of Colombia from 2013 to 2023 in 5-minutes intervals (Original data)
